# Altered Activity of SK Channel Underpins Morphine Withdrawal Relevant Psychiatric Deficiency in Infralimbic to Accumbens Shell Pathway

**DOI:** 10.3389/fpsyt.2019.00240

**Published:** 2019-04-11

**Authors:** Liang Qu, Yuan Wang, Shun-Nan Ge, Nan Li, Jian Fu, Yue Zhang, Xin Wang, Jiang-Peng Jing, Yang Li, Qiang Wang, Guo-Dong Gao, Shi-Ming He, Xue-Lian Wang

**Affiliations:** Department of Neurosurgery, Tangdu Hospital, The Fourth Military Medical University, Xi’an, China

**Keywords:** morphine, conditioned place preference, nucleus accumbens, medium spiny neurons, medial prefrontal cortex, small conductance calcium-activated potassium channels (SK channels)

## Abstract

Drug addiction can be viewed as a chronic psychiatric disorder that is related to dysfunction of neural circuits, including reward deficits, stress surfeits, craving changes, and compromised executive function. The nucleus accumbens (NAc) plays a crucial role in regulating craving and relapse, while the medial prefrontal cortex (mPFC) represents a higher cortex projecting into the NAc that is active in the management of executive function. In this study, we investigated the role of the small conductance calcium-activated potassium channels (SK channels) in NAc and mPFC after morphine withdrawal. Action potential (AP) firing of neurons in the NAc shell was enhanced *via* the downregulations of the SK channels after morphine withdrawal. Furthermore, the expression of SK2 and SK3 subunits in the NAc was significantly reduced after 3 weeks of morphine withdrawal, but was not altered in the dorsal striatum. In mPFC, the SK channel subunits were differentially expressed. To be specific, the expression of SK3 was upregulated, while the expression of SK2 was unchanged. Furthermore, the AP firing in layer 5 pyramidal neurons of the infralimbic (IL) cortex was decreased *via* the upregulations of the SK channel-related tail current after 3 weeks of morphine withdrawal. These results suggest that the SK channel plays a specific role in reward circuits following morphine exposure and a period of drug withdrawal, making it a potential target for the prevention of relapse.

## Introduction

Drug addiction can be viewed as a mental health disorder caused by maladaptive neural plasticity (1). It involves long-term and persistent dysregulation of neural circuits, particularly in motivational systems and reward systems (2). Opioids, including morphine, are the first-line choice for the management of chronic pain and moderate-to-severe acute pain in both cancer and noncancer patients (3). However, repeated morphine exposure can lead to addiction, and relapse after morphine withdrawal occurs easily in the majority of individuals (3–5). Furthermore, increasingly higher doses of morphine are frequently used to overcome tolerance. This stratagem also exposes patients to a higher risk of developing severe adverse reactions and side effects in the reward circuitry and can lead to withdrawal symptoms (6, 7).

The ventral tegmental area (VTA)–nucleus accumbens (NAc) circuit plays a crucial function in drug reward processing (8–10). The general understanding of the reward circuitry originates with the VTA, which is composed of 60–65% dopaminergic neurons (11, 12). Moreover, a previous study has shown that practically all known abused drugs increase DA release in the NAc core or shell, which exerts its effects *via* the activation of DA receptors located on medium spiny neurons (MSNs) (13). The NAc region of the ventral striatum is a main part of the reward circuitry, and is of great importance for various aspects of addiction, such as relapse and craving (9). MSNs are the predominant cell type in both the shell and core regions of NAc, and have specific features including spiny morphology, low basal ﬁring rates *in vivo*, and lack of spontaneous activity *in vitro* (6, 14). MSNs in the NAc core appear to be indispensable for assigning motivational values to discrete stimuli associated with reward or aversion, and particularly to renewing these values as environments change (15). MSNs in the NAc shell drive behavioral reactions to repeated exposure to rewarding experiences, such as chronic opioid administration (15). The prelimbic (PL) and infralimbic (IL) cortices, the two subregions of the medial prefrontal cortex (mPFC), play essential roles in the neural circuits for memory extinction and influence the extinction of drug memories, thus reducing the risk of subsequent relapse (16, 17). While the PL projects to both the core and shell of NAc, the IL projects to the shell preferentially (18, 19).

Action potential (AP) firing plays a foremost role in the mechanism of storing and processing information by changing the strength of connections between neurons (20). The generation of signals in NAc core and shell neurons is believed to be related to certain behavioral events, such as the activation of primary reward, motivation, reinstatement/relapse induced by opioid exposure, and several categories of drug-related cues, together with several classes of conditioning occurrences (21–24). In neurons, small conductance calcium-activated potassium channels (SK channels) influence somatic excitability by contributing to afterhyperpolarization and modulate synaptic plasticity by coupling to N-Methyl-D-aspartate (NMDA) receptors (25). There are two homologous SK channels (SK2 and SK3) expressed in striatal neurons (24, 26). SK channels are gated solely by intracellular Ca^2+^ ions, and the activity of SK channel is regulated by protein phosphatase 2A (PP2A) and protein kinase CK2 (CK2) (27).

Numerous lines of evidence indicate that the activated opioid receptors in VTA contribute to the effects of morphine in NAc (28–31). Several studies have focused on the NAc core and shell firing during short-term morphine withdrawal *in vivo* (32, 33). A recent report demonstrated that the molecular neuro-adaptations in the NAc core and lateral dorsal striatum could potently enhance drug-seeking activity (34). In addition, the reduction in the function of the SK channel, which increases tonic firing in the NAc core after long-term alcohol self-administration and protracted abstinence, may indicate that it is a critical regulator of motivation after abstinence (34–36). However, it is still unclear whether the alterations of neuronal firing could persist after long-term morphine withdrawal, and whether it could change the expression or activity of the SK channel in these regions.

Our previous study demonstrated that the ability of the transient receptor potential vanilloid 1 (TRPV1) to regulate excitatory glutamatergic transmission in the NAc is enhanced during morphine withdrawal (37). To further comprehend the significance of these neuro-adaptations for morphine withdrawal, we assessed the effect of long-term morphine withdrawal on the SK channel in the NAc and mPFC. In this study, we investigated whether morphine withdrawal can alter the expression or activity of subtypes of SK channels in the NAc core and shell (ventral striatum) and mPFC during the withdrawal period. Our study linked SK channels induced electrophysiological changes in a specific brain region to an increase in drug-seeking behavior.

## Materials and Methods

### Animals

Sixty Sprague–Dawley rats (male, 150–250 g body weight) were obtained from the Animal Care Committee of the Fourth Military Medical University (FMMU) (Xi’an, China). All experimental methods and procedures were carried out in compliance with the guidelines and regulations of Animal Care and Use Committee, FMMU, and this study was approved by the institutional ethical committee of Tangdu hospital, FMMU (Approval No. 2017LCYJ002). The rats were individually housed in cages at standard humidity (approximately 50%) and room temperature (20–23°C) with a 12-h light–12-h dark cycle (lights on at 8:00 AM). All tests were performed during the light phase. The animals were allowed to habituate to the laboratory conditions for 7 days before the beginning of conditioned place preference (CPP) pretest.

### Drugs

Morphine was obtained from Shenyang No. 1 Medical Drugs Company (Shenyang, China) and saline was acquired from Disai Biological Pharmaceutical Company (Xi’an, China). Saline (0.9%) or morphine (10 mg·kg^−1^) administrations were provided as subcutaneous (s.c). injections for seven consecutive days followed by 3 weeks of long-term withdrawal. The protocol of morphine administration was based on previous literature with CPP (37–39).

### Conditioned Place Preference Procedure

CPP was carried out fundamentally as described previously (38, 39). Rats were trained to acquire CPP in the conditioning apparatus (Noldus Information Technology Co., Ltd, Netherlands). Briefly, the CPP training consisted of three different parts. 1) CPP pretest (day 1): the rats were individually placed in the intermediate compartment and allowed to move freely for 900 s in all compartments. Rats were excluded from the study for having an unconditioned side bias if they spent more than two-thirds of the total time in one of the compartments. 2) CPP conditioning period (days 2–8): morphine (10 mg·kg^−1^) and saline were administered on alternating days to the morphine-treated CPP rats, and they were immediately placed in the least preferred compartment for 45 min. 3) CPP test (day 9): the rat had access freely to all compartments for 900 s. The “preference time score” (PTS) was termed as time spent in the drug-paired compartments minus the time spent in the saline-paired compartments. The movement of each rat was recorded using a video camera on top of the compartments, and total time spent in each of the conditioning compartments was measured using ETHOVISION 3.1 software (Noldus Information Technology Co., Ltd., Netherlands).

### Slice Preparation and *Ex Vivo* Electrophysiology

Rats from saline-treated groups or morphine-treated groups were anaesthetized with an intraperitoneal injection of chloral hydrate (15 mg·kg^−1^, Aoxin Chemical Factory, Yangzhou, China) and decapitated. The brains were rapidly removed and submerged in ice-cold modified artificial cerebrospinal fluid (ACSF) containing 225 mM sucrose; 119 mM NaCl, 2.5 mM KCl, 4.9 mM MgCl_2_, 0.1 mM CaCl_2_, 26.2 mM NaHCO_3_, 1.0 mM NaH_2_PO_4_, 1.25 mM glucose; 1 mM ascorbic acid; and 3 mM kynurenic acid. The brain was removed rapidly, and coronal slices (250–300 µm) containing the NAc shell or IL cortex were cut in the same modified ACSF as described previously (38). The slices were recovered at 32°C in carbogen-bubbled ACSF comprising 126 mM NaCl, 2.5 mM KCl, 1.2 mM MgCl_2_, 2.4 mM CaCl_2_, 18 mM NaHCO_3_, 1.2 mM NaH_2_PO_4_, and 11 mM glucose, with pH 7.2–7.4 and 301–305 mOsm. During the trials, the slices were submerged and continuously perfused with carbogen-bubbled ACSF warmed to 32°C, and picrotoxin (50 μM; Sigma, USA) and CNQX (10 μM; Sigma, USA) were added to block GABA receptors and AMPA-type glutamate receptors. The trials were restricted to the GABAergic MSNs, which comprise more than 90% of the efferent neurons within the NAc core and shell, while other cell types can easily be set apart by a large soma or by very high rates of firing and a larger after hyperpolarization potential (AHP) (40–42).

Whole-cell recordings were attained using a Multiclamp 700B amplifier (Axon Instr., USA). Neurons were patched with a 3–5 MΩ micropipette, which was pulled using a P-97 puller (Sutter Instr., USA). The intracellular solution contained 130 mM KOH, 2.8 mM NaCl, 17 mM HCl, 20 mM HEPES, 105 mM methane sulfonic acid, 0.3 mM EGTA, 2.5 mM MgATP, 0.25 mM GTP, pH 7.2–7.4, 275–285 mOsm. A low level of the calcium-buffering agent EGTA was included in the pipette solution in order to sustain calcium-dependent potassium currents during whole-cell current and voltage clamp recordings (34). To measure firing, current pulses were applied using a patch amplifier in current clamp mode, and a sequence of seven to eight current pulses (300-ms duration, 20 pA apart) were applied every 30 s. The minimum current amplitude was adjusted for each neuron so that the first pulse was just subthreshold for spike firing. Depolarizing pulses were interspersed with a 33.3-pA hyperpolarizing pulse to examine the input resistance. Using the anterior commissure (AC), the lateral ventricles, and the dorsal striatum as landmarks, the NAc shell and IL cortex could be readily found in the experiments of patch clamping. The shape of NAc shell in coronal section is approximately like a ring. The distance from NAc shell to AC is about 4–13 mm. Individual neurons from NAc Shell or layer 5 pyramidal cells located in the IL subregion of the mPFC were visually identified using an upright infrared differential interference contrast microscope (BX51WI; Olympus, Japan).

### Western Blotting

The protein expression of the SK2 and SK3 subunits was assayed in the NAc core and shell (ventral striatum) during drug withdrawal as described previously (26, 34, 43). Lysis buffer contained 50 mM Tris-HCl, 150 mM NaCl, 1% Triton X-100, 0.5% sodium deoxycholate, 0.1% sodium dodecyl sulfate, pH 8.0, supplemented with 1% protease inhibitor cocktail (P8340; Sigma Aldrich, USA). Antibodies used were SK2 C-terminus (cat. # APC-045, 1:800, Alomone, Jerusalem, Israel), SK3 N-terminus (cat. # APC-025, 1:800, Alomone, Jerusalem, Israel), β-actin (1:5,000, TA-09; ZSGB-BIO Co., Beijing, China). Fresh samples were lysed in lysis buffer (previously mentioned). Then, the protein concentration was determined using a bicinchoninic assay kit (Beyotime, Ltd., Haimen, China) according to the kit manufacturer’s protocol. Equal quantities of protein from the NAc core and shell, dorsal striatum, or mPFC were resolved on 8% acrylamide SDS-PAGE gels and electrophoretically transferred to polyvinylidene fluoride (PVDF) membranes (Millipore, Billerica, MA, USA). The membranes were blocked for 2 h in 5% skim milk diluted in PBS/tween [PBS containing Triton X-100 (PBST), 0.01 M PBS with 0.1% Tween 20] at 37°C with gentle shaking. The membranes were then incubated overnight with antibodies reactive against the SK2 C-terminus or SK3 N-terminus, overnight at 4°C in 4% skim milk. After being washed in PBST, the membranes were incubated with the HRP-conjugated secondary antibodies. Blots were developed with chemiluminescence (Chemi Doc XRS+; Bio-Rad, CA, USA). Band intensity was quantified and analyzed using ImageJ 4.0 (National Institutes of Health, Bethesda, MD, USA), and the expression of SK2 and SK3 was normalized to that of β-actin (1:5,000, TA-09; ZSGB-BIO Co., Beijing, China).

### Immunohistochemistry

The animals were deeply anesthetized with an i.p. injection (15 mg·kg^−1^) of chloral hydrate (Aoxin Chemical Factory, Yangzhou, China) and transcardially perfused with 100 ml of PBS, followed by 100 ml of PBS with 4% paraformaldehyde (PFA; Sigma-Aldrich, St. Louis, USA), pH 7.4. Brain tissues were carefully dissected, postfixed overnight with 4% PFA at 4°C, and cut into 30-μm sections using a vibratome (VT1000S; Leica, Wetzlar, Germany). For SK3 and NeuN immunostaining, sections were washed in 0.1 M phosphate buffer. The slices were then incubated in PBS with 0.2% Triton X-100 for 10 min, followed by washing with PBS (3×5 min), blocked in 1% normal horse serum in 0.1 M phosphate buffer for 30 min at room temperature, and subsequently incubated overnight at 4°C with the following primary antibodies: mouse monoclonal anti-NeuN (cat. # MAB377, 1:1,000; Millipore, Billerica, USA) and rabbit anti-SK3 monoclonal antibody (cat. # APC-025, 1:500, Alomone, Jerusalem, Israel) in PBS. Following washing in PBS (3×5 min), Cy2-conjugated anti-mouse IgG (1:200, Jackson ImmunoResearch Laboratories, USA) and Cy3-conjugated anti-rabbit IgG (1:200, Jackson ImmunoResearch Laboratories, USA) were used for fluorescence detection. Nuclei counterstaining was performed using 4’,6-diamidino-2-phenylindole dihydrochloride (DAPI; cat. D9542, Sigma). Fluorescence images were captured using a confocal microscope (A1; Nikon, Japan).

### Statistical Analysis

The results of *ex vivo* electrophysiological recording were analyzed using pClamp 10.2 software (Axon Instr., USA) and Origin 9.0 software (Origin Lab, Northampton, MA, USA). For the reason that a different number of neurons was recorded for each rat, baseline spike firing and voltage clamp parameters (including baseline input/output slope, AP waveform and input resistance parameters, and tail currents) were averaged for all neurons achieved from a given animal, thus acquiring a specific value of each of these parameters for each individual rat. The data are expressed as means ± SEM for all the tests. Statistical analysis of results was presented using paired *t*-tests and ANOVA for the data of influences of morphine withdrawal on neuronal firing and AHP currents. Unless otherwise noted, all statistics were presented using a two-way repeated-measures ANOVA (RM-ANOVA). All tests of statistical significance were two-sided and the threshold of statistical significance was set at *p* < 0.05.

## Results

### Nucleus Accumbens Shell Action Potential Firing was Enhanced After Morphine Withdrawal

**Figure 1:**
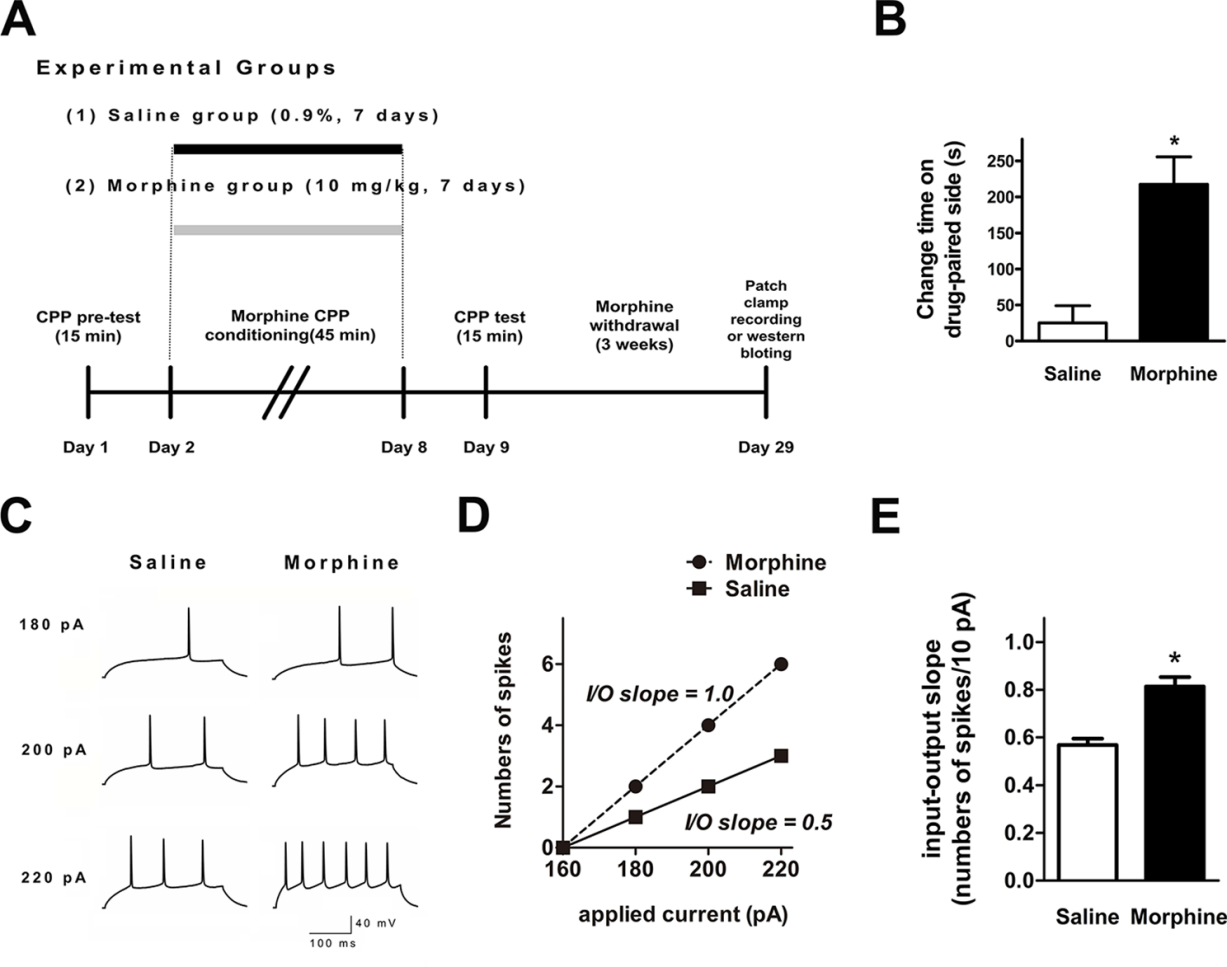
Spike firing in the nucleus accumbens (NAc) shell was significantly enhanced after 3 weeks of morphine withdrawal. **(A)** Morphine-induced conditioned place preference of experimental groups over time. All animals were sacrificed 3 weeks after the final conditioned place preference (CPP) test. **(B)** For the CPP, the difference in the time spent in the less-preferred and drug-paired compartment between the preconditioning and postconditioning phase (n = 12). *p < 0.05 compared with the saline group. **(C)** Example traces of action potential (AP) generation evoked in response to depolarizing current steps in NAc shell neurons from saline mock-treated rats or morphine withdrawal rats. **(D)** Example input/output relationships (I/O slope) derived from the saline mock treatment group and morphine withdrawal group traces in (C). **(E)** Grouped data showing enhanced spike firing in NAc shell neurons from the saline mock treatment group versus morphine withdrawal group. The data are shown as means ± S.E.M., *p < 0.05 vs. saline.

We performed *ex vivo* whole-cell patch-clamp recordings to evaluate whether NAc shell AP firing was changed after 3 weeks of withdrawal from seven consecutive days of morphine administration (10 mg·kg^−1^) *via* subcutaneous injections (**Figure 1A**). **Figure 1B** indicates that the morphine-treated group spent a significantly longer time in the drug-paired compartment, compared to the saline-treated group (t = 4.273, *p* = 0.0003, n = 12). *Ex vivo* electrophysiological experiments were carried out in current-clamp mode, where 300-ms depolarizing current pulses (both sub- and suprathreshold for firing) were used to elicit AP firing (**Figure 1C**). The resting membrane potential of each neuron was set to –90 mV before provoking the firing. The number of APs was significantly increased in NAc shell neurons after 3 weeks of withdrawal from morphine injection (**Figure 1C and D**). In order to describe the input/output relationship between spike firing and a series of depolarizing current pulses, we used the ‘‘input/output slope’’ (I/O slope) to analyze the data as done in a previous study (34). The slope was calculated from the number of spikes generated in the last subthreshold current pulse and the first three suprathreshold current pulses. NAc shell neurons from morphine-treated rats exhibited a significantly larger basal I/O slope than neurons from saline-treated rats (**Figure 1E**, saline: n = 12, 0.57 ± 0.03 AP/10 pA; morphine: n = 18, 0.81 ± 0.04 AP/10 pA, t = 5.176, *p* = 0.0001).

### SK Inhibition Differentially Enhanced Firing in Nucleus Accumbens Shell Neurons From Morphine and Saline Control Rats

**Figure 2:**
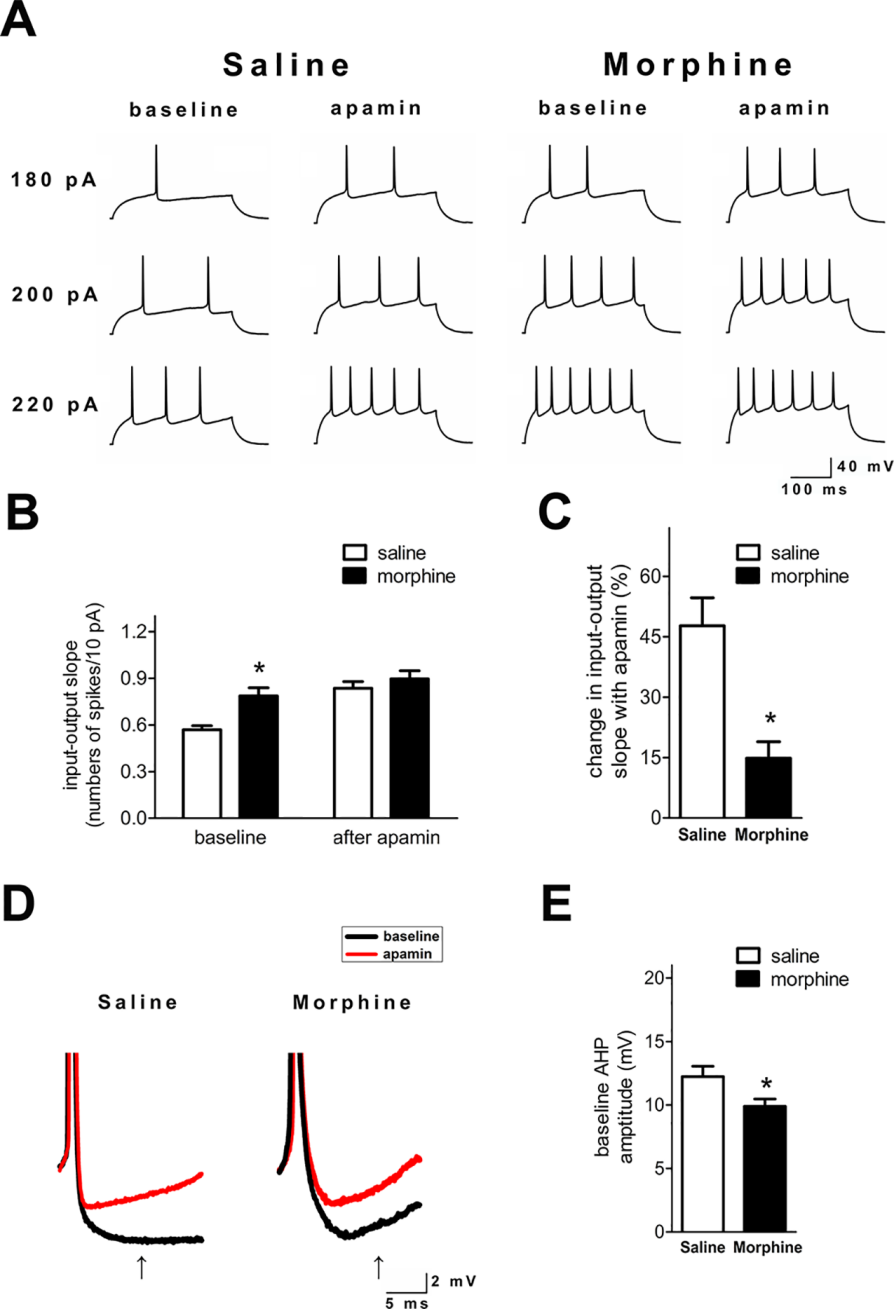
SK inhibition enhanced neuronal firing in the NAc shell after 3 weeks of morphine withdrawal. **(A)** Examples illustrating that SK inhibition produced a greater enhancement of the firing rate in neurons from the saline mock treatment group versus the morphine withdrawal group. **(B)** Grouped data showing the changes of input/output slopes after apamin addition in both groups. **(C)** The relative proportions of apamin-induced changes in the I/O slope were significantly greater in neurons from the saline mock treatment group than those from the morphine withdrawal group. **(D)** Magnification of the AHP in **(A)** illustrating the amplitude of the AHP threshold at 15 ms after the AP threshold (arrow). **(E)** Grouped data showing that the amplitude of the AHP was significantly reduced in neurons from the morphine withdrawal group versus the saline mock treatment group. The data are presented as means ± S.E.M., *p < 0.05 vs. saline.

SK inhibition with apamin enhanced the *ex vivo* NAc shell firing in both groups (**Figure 2A**). However, the I/O slope was significantly greater in NAc shell neurons from morphine withdrawal rats, but there were no significant differences between the two groups after exposure to apamin (**Figure 2B and C**; saline control: n = 14 from 10 rats; morphine withdrawal: n = 12 from 10 rats; apamin: F_(1,36)_ = 9.818, *p* = 0.004; group: F_(1,36)_ = 17.89, *p* < 0.001; apamin x group: F_(1,36)_ = 6.336, *p* = 0.085; two-way RM-ANOVA; *p < 0.05 morphine withdrawal group versus saline control group before apamin). Thus, SK inhibition by apamin eliminated the difference in the I/O slopes of the two groups after apamin exposure, which suggests that basal alterations in firing reflect differential SK function. Specifically, a decrease in basal SK currents could enhance the neuronal excitability in rats after morphine withdrawal. Moreover, the magnitude of the slower component of the AHP at 15 ms after the AP threshold was significantly decreased in NAc shell neurons from the morphine withdrawal animals versus the saline control animals at baseline (**Figure 2D and E**; t = 2.334, *p* = 0.035; *p < 0.05 morphine withdrawal versus saline control).

### SK Currents in the Nucleus Accumbens Shell Were Reduced After Morphine Withdrawal

**Figure 3:**
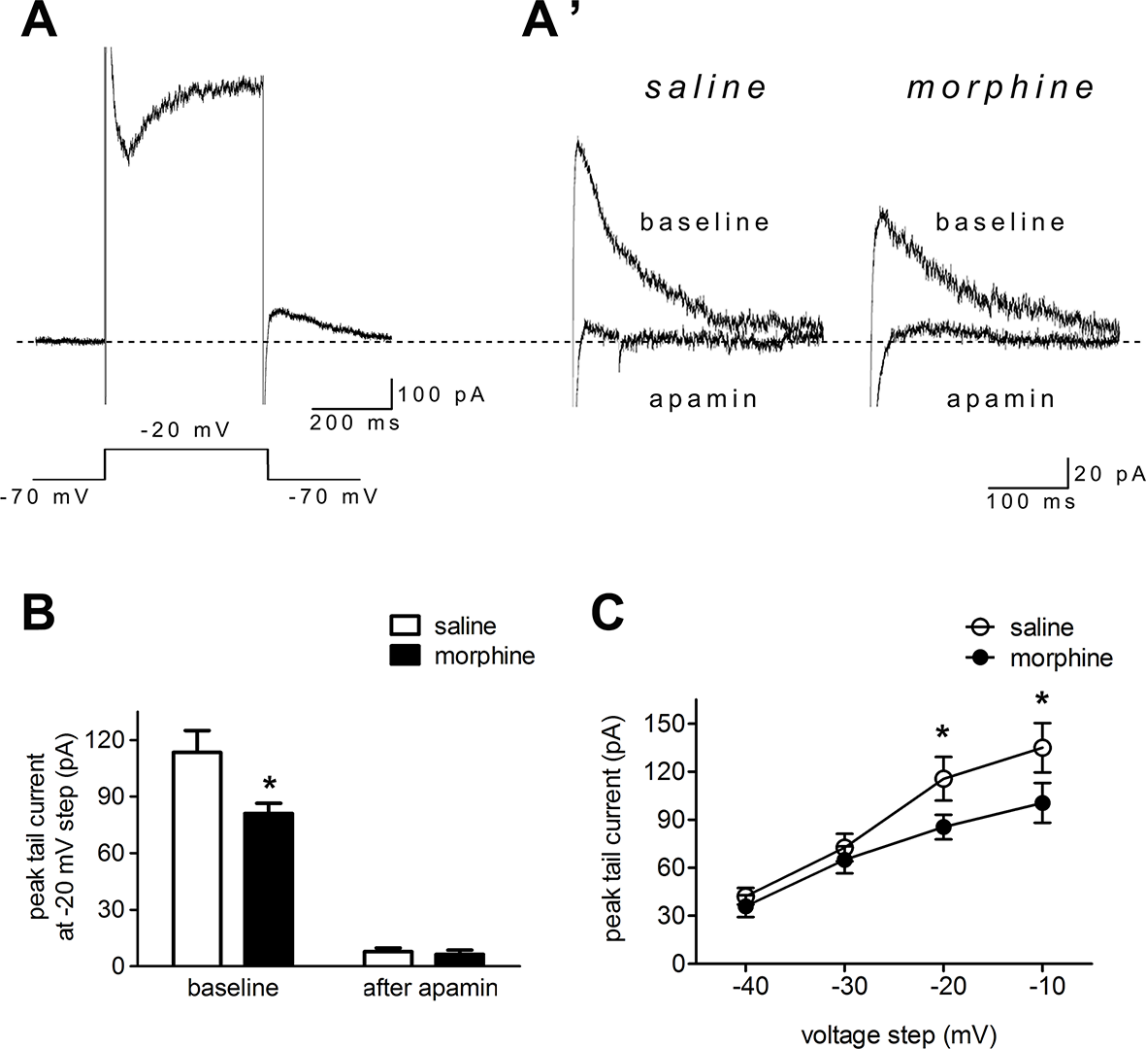
Morphine withdrawal decreased SK currents in NAc shell neurons. **(A)** An example of an entire current response upon depolarization to –20 mV from a –70-mV holding potential in voltage clamp mode, with an apparent tail current after returning to –70 mV following the depolarization; **(A’)** magnified example tail currents. **(B)** Grouped data showing that peak tail currents were reduced in neurons from the morphine withdrawal group versus the saline mock treatment group. **(C)** Peak tail currents (induced by depolarization to –40 to –10 mV) were significantly different in neurons from the morphine withdrawal group than in those from the saline mock treatment group. The data are shown as means ± S.E.M., *p < 0.05 vs. saline.

To directly test NAc shell SK function after morphine withdrawal, we used voltage clamp methods to isolate SK currents (34, 44). The neurons were held at –70 mV, then depolarized for 400 ms to voltages ranging from –40 to –10 mV (in 10-mV steps) prior to being brought back to –70 mV. A tail current was evident upon returning to –70 mV (**Figure 3A and A’**), which may reflect slow ion channel deactivation. Peak tail currents were significantly smaller in NAc shell neurons from the morphine withdrawal animals than in those from saline-treated controls, and SK inhibition with apamin nearly abolished the tail current in both groups (**Figure 3A’ and B**; saline control group: n = 12 from 8 rats; morphine withdrawal group: n = 14 from 11 rats; baseline: saline 113.5± 16.2 pA, morphine 81.1 ± 8.2 pA; after apamin: saline 7.9 ± 2.0 pA, morphine 6.3 ± 2.2 pA; apamin: F_(1,34)_ = 3.98, *p* = 0.054; group: F_(1,34)_ = 112.14, *p* < 0.001; apamin x group: F_(1,34)_ = 3.27, *p* = 0.0795; two-way RM-ANOVA; *p < 0.05 morphine withdrawal group versus saline control group before apamin). These imply that the peak tail current can be largely seen as SK-mediated currents (34, 44). Average basal tail current peak amplitudes at voltages from -40 to -10 mv in the saline control group were 42.2 ± 5.2, 36.0 ± 6.8A, 72.6 ± 8.6, and 65.1 ± 8.4 pA, and those in the morphine withdrawal group were 115.6 ± 13.6, 85.4 ± 7.6, 135.3 ± 15.4, and 100.5 ± 12.5 pA, respectively (**Figure 3C**; n = 16 for saline control, n = 20 for morphine withdrawal; voltage: F_(1,136)_ = 131.78, *p* < 0.001; group: F_(3,136)_ = 414.49, *p* < 0.001; voltage × group: F_(3,136)_ = 18.76, *p* < 0.001; two-way RM-ANOVA; *p < 0.05 morphine withdrawal group versus saline control group).

### Protein Expression of the SK2 and SK3 Subunits was Changed in Both the Medial Prefrontal Cortex and Nucleus Accumbens Core and Shell During Morphine Withdrawal

**Figure 4:**
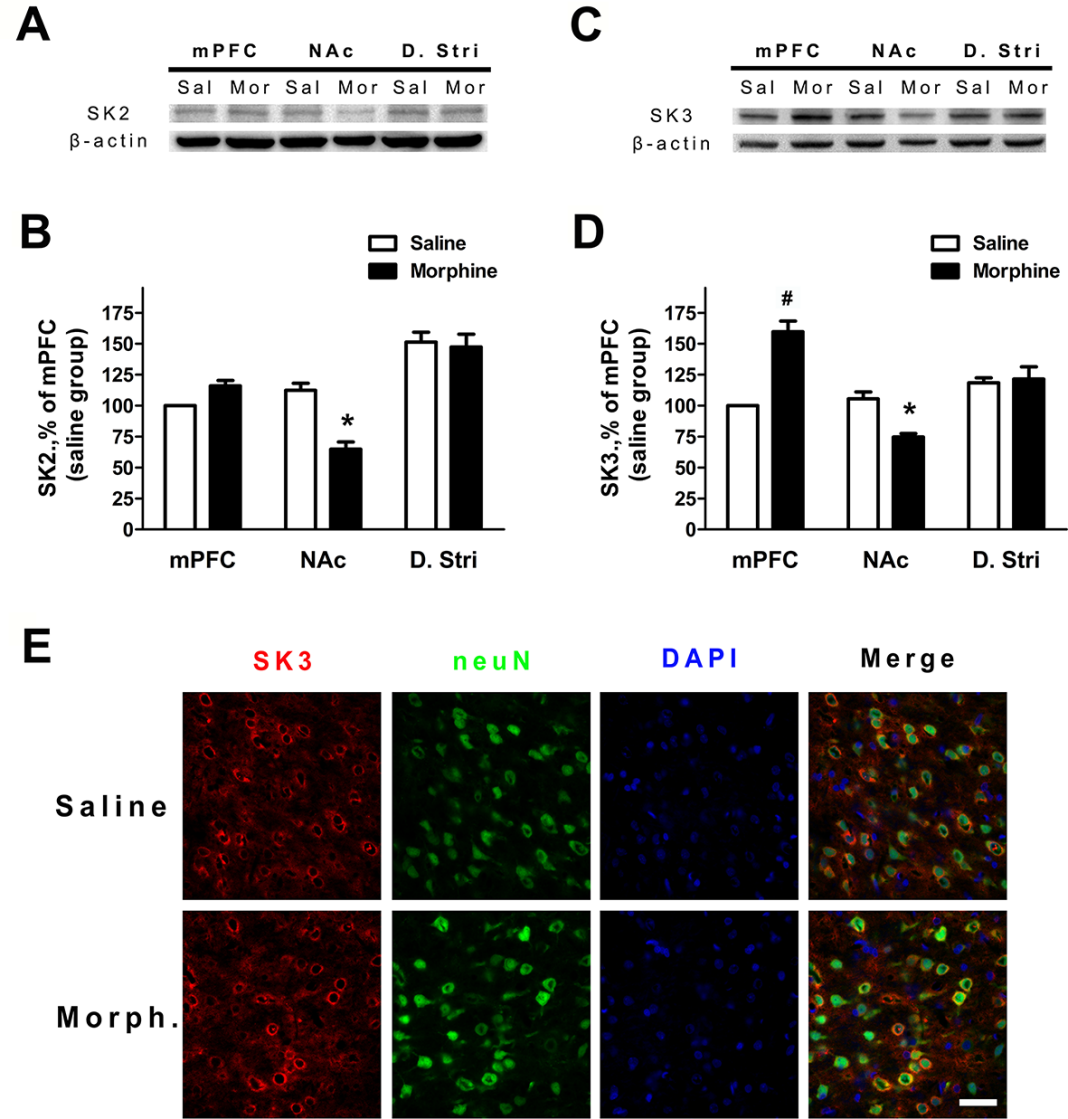
The protein expression of SK2 and SK3 subunits was changed in different brain regions after 3 weeks of morphine withdrawal. **(A)** Representative Western blots showing the changes of SK2 subunit protein expression in mPFC, NAc, and dorsal striatum after morphine withdrawal. **(B)** Quantitative analysis of SK2 subunit protein expression in **(A)**, normalized to β-actin. The data are shown as means ± S.E.M., *p < 0.05 vs. saline. **(C)** Representative Western blots showing the changes SK3 subunit protein expression in mPFC, NAc, and dorsal striatum after morphine withdrawal. **(D)** Quantitative analysis of SK3 subunit protein expression in **(C)**, normalized to β-actin. The data are shown as means ± S.E.M., *p < 0.05 vs. saline. (E) SK3/NeuN/DAPI-positive neurons in the infralimbic (IL) cortex after 3 weeks of morphine withdrawal; scale bar = 50 μm.

To verify whether the expression of SK channels in the mPFC and NAc core and shell (ventral striatum) changed during morphine withdrawal, we examine the expression of its SK2 and SK3 subunits, which are most abundant in the mPFC, NAc core and shell, and dorsal striatum (24, 26, 34). The results demonstrated that SK2 subunit expression was significantly decreased in the NAc core and shell of the morphine withdrawal rats compared to that of saline control rats (**Figure 4A and B**; saline: n = 12; morphine: n = 12; location: F_(1,66)_ = 5.05, *p* = 0.0442; group: F_(2,66)_ = 45.56, *p* < 0.001; location × group: F_(2,66)_ = 12.53, *p* = 0.0012; two-way RM-ANOVA, *p < 0.05 morphine withdrawal versus saline control in NAc). In addition, no changes of protein expression of the SK2 subunit were observed in the mPFC and dorsal striatum (**Figure 4A and B**). Interestingly, the results showed that the expression of SK3 was significantly increased in the mPFC and reduced in the NAc core and shell of morphine withdrawal rats compared to that of saline control rats (**Figure 4C and D**; saline: n = 10; morphine: n = 12; location: F_(1,60)_ = 4.43, *p* = 0.0570; group: F_(2,60)_ = 22.57, *p* < 0.001; location × group: F_(2,60)_ = 27.65, *p* < 0.001; two-way RM-ANOVA, *p < 0.05 morphine withdrawal versus saline control, ^#^ *p* < 0.001 morphine withdrawal versus saline control in mPFC, *p < 0.05 morphine withdrawal versus saline control in NAc). Thus, morphine withdrawal was associated with enhanced protein expression of the SK3 but not the SK2 subunit in the mPFC, suggesting that enhanced SK3 expression likely contributed to the observed increase in SK currents after morphine withdrawal. **Figure 4E** shows the SK3/neuN immunostaining of layer 5 pyramidal neurons in the IL, which preferentially projects to the NAc shell (**Figure 4E**; saline control group and morphine withdrawal group). These data show there are a high proportion of SK3 positive neurons in Layer 5 of IL after morphine withdrawal or saline control.

### Action Potential Firing and SK Currents Were Changed in the Infralimbic Cortex After Morphine Withdrawal

**Figure 5:**
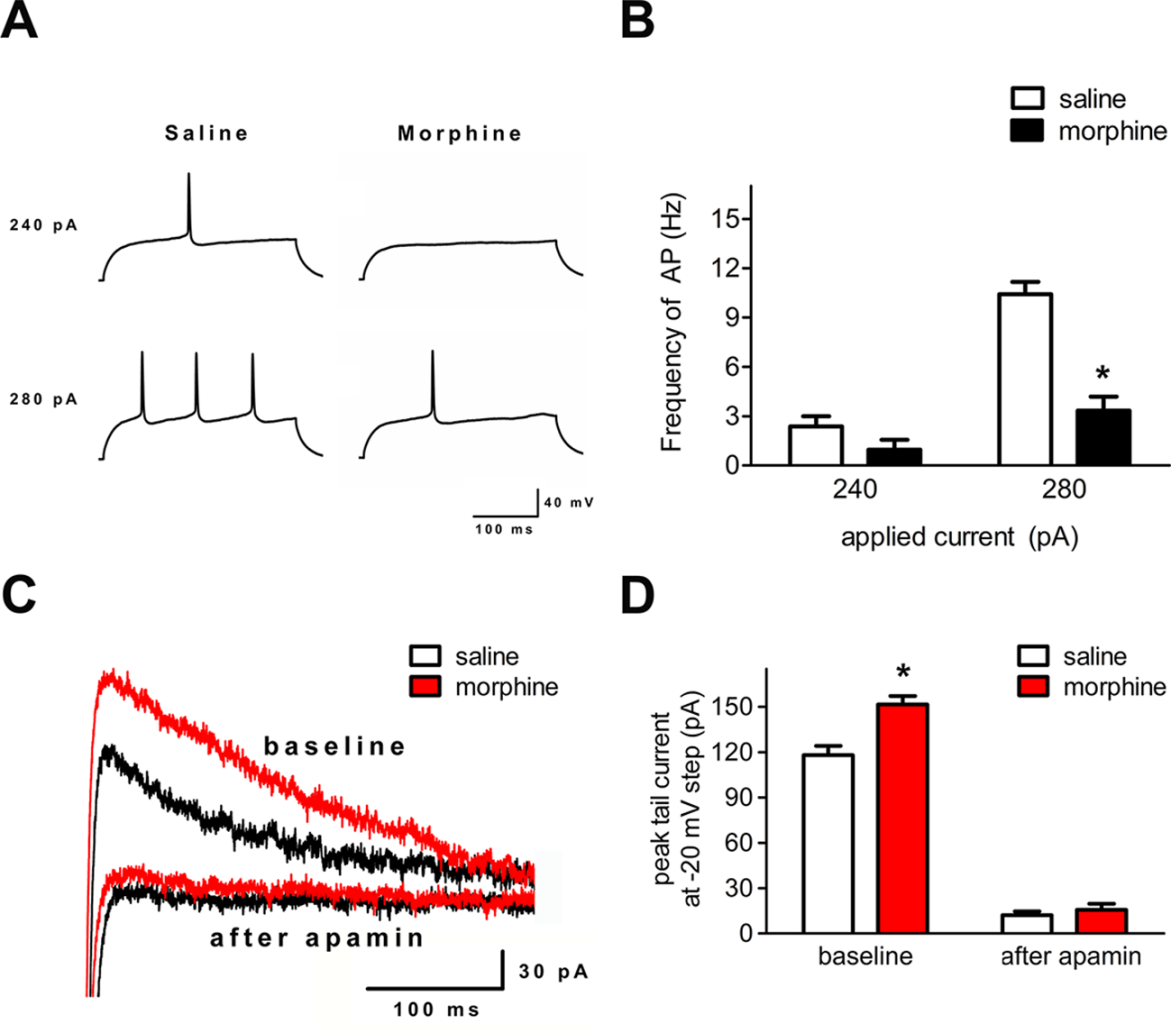
Spike firing and SK currents were changed in layer 5 pyramidal neurons of the IL cortex after 3 weeks of morphine withdrawal. **(A)** Example traces of AP generation evoked in response to depolarizing current steps in pyramidal neurons from saline mock treatment rats or morphine withdrawal rats. **(B)** Grouped data showing reduced spike firing in neurons after 3 weeks of morphine withdrawal. The data are shown as means ± S.E.M., *p < 0.05 vs. saline. **(C)** Examples of magnified tail currents induced by depolarized to –20 mV from a –70-mV holding potential, after returning to –70 mV following the depolarization. **(D)** Grouped data showing that peak tail currents were increased in neurons from the morphine withdrawal group versus the saline mock treatment group.

To verify whether the neuronal excitability and the function of the SK channel changed in layer 5 pyramidal neurons of the IL cortex after morphine withdrawal, electrophysiological experiments were performed. In current-clamp mode, depolarizing current pulses (300 ms, 240–280 pA) were applied to elicit AP firing (**Figure 5A**). The number of APs was significantly decreased in IL neurons (**Figure 5A and B**, saline control group: n = 18 from 14 rats; morphine withdrawal group: n = 21 from 14 rats current: F_(1,52)_ = 35.10, *p* < 0.001; group: F_(1,52)_ = 52.54, *p* < 0.001; current × group: F_(1,52)_ = 15.51, *p* < 0.001; two-way RM-ANOVA, *p < 0.05 morphine withdrawal group versus saline control group). Peak tail currents were significantly larger in IL neurons from the morphine withdrawal group than in those from the saline control group (**Figure 5C and D**; saline control group: n = 15 from 9 rats; morphine withdrawal group: n = 18 from 12 rats; baseline: saline 118.5 ± 14.1 pA, morphine 151.6 ± 10.2 pA; after apamin: saline 12.2 ± 3.4 pA, morphine 15.7 ± 4.9 pA; apamin: F_(1,38)_ = 15.27, *p* = 0.0175; group: F_(1,38)_ = 643.43, *p* < 0.001; apamin × group: F_(1,38)_ = 9.94, *p* = 0.0344; two-way RM-ANOVA, *p < 0.05 morphine withdrawal group versus saline control group).

## Discussion

Our results show that chronic morphine withdrawal increases the intrinsic neuronal excitability of MSNs in the NAc shell. We identified a relationship between neuro-adaption and SK channels. Our data also showed that the increased AP firing was mediated by the slow component of the afterhyperpolarization current, which could be eliminated using the SK channel antagonist apamin. Furthermore, we investigated the protein expression of SK channel subunits in the NAc and mPFC. The data demonstrated that the expression of the SK2 and SK3 subunits was significantly reduced in the NAc after 3 weeks of morphine withdrawal, while it was not altered in the dorsal striatum. We further investigated the expression and function of SK channels in the mPFC, and the data showed that morphine withdrawal decreased the intrinsic excitability and SK current in layer 5 pyramidal neurons of the IL cortex.

Drug addiction represents a dramatic dysregulation of neural circuits that lead to reward deficits and stress surfeits, craving changes during the reward or stress process, and compromised executive function (45). Dopaminergic neurons located in the VTA and projecting to the NAc play a key role in the processing of reward-related stimuli, including those associated with drug abuse (46). The changes of craving and deficits in executive function involve the dysregulation of crucial afferent projections from the prefrontal cortex (PFC) and insula to the basal ganglia and extended amygdala (47, 48). The medial PFC plays an important role in the higher-order executive processes and sends highly organized projections to subcortical regions controlling motivation (49). A previous study showed that chronic withdrawal from repeated morphine exposure elicits potentiation in both glutamatergic synaptic strength and intrinsic excitability of MSNs in the NAc shell (50). Moreover, a recent study reported that morphine-induced plasticity changes in IL cortex–NAc shell projections could regulate the reinstatement of morphine-evoked CPP (51). Our data confirmed that the I/O slope, which reflects the intrinsic excitability of the NAc shell, was enhanced after 3 weeks of morphine withdrawal (**Figure 1E**), and showed that AP firing and SK currents of the layer 5 pyramidal neurons in the IL cortex were changed after morphine withdrawal (**Figure 5B and D**). These findings confirm the presence of distinct neuronal changes in the IL cortex–NAc shell projections that may provide targetable molecular mechanisms for future pharmacotherapies. Furthermore, these observations may be evidence of a relationship between morphine-induced dysfunction of the higher-order executive processes in mPFC and morphine-induced changes of craving-related signals in the NAc.

Several studies have pointed out that numerous ion channel mechanisms are involved in opioid exposure or withdrawal (52–55). Our own previous study demonstrated that the ability of TRPV1 to regulate excitatory glutamatergic transmission in the NAc is enhanced during morphine withdrawal (37). Cognitive inhibition of craving is one of the cognitive control techniques involved in the higher-order executive function of the mPFC that enhance the patient’s ability to cope with cravings and prevent relapse (56). Motivations or emotions generated by subcortical circuits involving the NAc may be powerfully modulated by the PFC (57, 58). Some studies also reported that mPFC inactivation also reduces morphine-induced DA release in the NAc, which regulates the neuronal excitability and function (59). Our results suggest that the decrease in SK channel function in the NAc shell after morphine withdrawal reflects the decreased levels of both SK2 and SK3 subunits. At the same time, the increased SK function in the mPFC after morphine withdrawal may be related to the enhanced levels of the SK3 subunit. Our study links the molecular changes in SK channel function in a particular brain region containing the reward and inhibition control circuitry, the NAc and mPFC, to dynamically balanced alterations of neuronal excitability during the drug-seeking period.

Opioids modulate the expression of genes involved in neuroplasticity through epigenetic changes and possible RNA modifications (54). Ultimately opioids perturb the intracellular signaling cascade and neural circuits, whose dysfunction is associated with long-term changes in craving (47). Several studies reported that the mRNA levels of voltage- and calcium-gated potassium channels increased in addicted rats (54). Previous evidence indicated that the changes of SK currents and neuronal excitability in the NAc represent a critical mechanism that facilitates the motivation to seek alcohol during abstinence (34). The data of the present study indicate that the protein expression of the SK2 and SK3 subunits was decreased in the NAc, while the protein expression of the SK3 subunit was increased in the mPFC after morphine withdrawal (**Figure 4B and D**). The present study thus adds more detailed information on the role of functional alterations in neuronal excitability and protein expression of SK channel subunits induced by morphine withdrawal. Moreover, our findings demonstrate that the function of the higher brain cortex, which participates in executive function, was altered due to a decrease in neuronal excitability *via* an increase in the SK current due to higher expression of the SK3 subunit.

Overall, the present findings offer new insights into the involvement of SK channel subunits in the NAc shell MSNs and layer 5 pyramidal neurons of IL cortex neurons. Understanding the molecular mechanisms active during morphine withdrawal is a crucial step on the path toward finding potential therapies for opioid relapse. Because ion-channel-mediated neuro-adaptation can facilitate drug-seeking behavior during morphine withdrawal, we explored the relationship between intrinsic excitability and SK function in the NAc and mPFC. For an optimal therapeutic strategy for addiction, all these factors and their interplay need to be taken into consideration. Further studies are needed to determine the pathophysiological role of SK channels in the process of reward and inhibition control.

## Ethics Statement

Male Sprague–Dawley rats were obtained from the Animal Care Committee of the Fourth Military Medical University (Xi’an, China). All experimental procedures were carried out in accordance with the Institutional Animal Care and Use Committee guidelines at the Fourth Military Medical University and had received ethical approval from the institutional ethical committee of Tangdu hospital, the Fourth Military Medical University (Approval No. 2017LCYJ002).

## Author Contributions

X-LW and LQ designed the study. SG and JF designed the behavioral paradigm. JF and YL collected data for the behavioral paradigm. LQ, YZ, QW, and XW processed the brain tissue samples. YW, YZ, and NL collected and analyzed the data. X-LW and SH interpreted the data. X-LW, LQ, and YW wrote and edited the manuscript. All authors critically reviewed the content and approved the final version for publication.

## Conflict of Interest Statement

The authors declare that the research was conducted in the absence of any commercial or financial relationships that could be construed as a potential conflict of interest.
